# Research as agitation: Generative activism in the age of resistance

**DOI:** 10.1371/journal.pgph.0000142

**Published:** 2022-01-04

**Authors:** Arnab Majumdar, Monica Mukerjee

**Affiliations:** Activists, Decolonise MSF; PLOS: Public Library of Science, UNITED STATES

A torrent of public attention has surfaced myriad allegations of exploitation, discrimination and abuse by humanitarian, development, international aid, and global agencies. Amid these, Médecins Sans Frontières (MSF), our former employer, has been thrust into the spotlight, owing in large part to the *témoignage* and activism of current and former employees mobilizing in a growing movement to “Decolonise MSF”. In June 2020, a staff-led open letter containing more than 1,100 signatures and 200 testimonies of abuse and discrimination went public [1]. MSF’s international leadership, in the aftermath, welcomed “the current debate on racism” but did not formally acknowledge the letter or its specific demands [2]. Since then, media attention has amplified insider concerns on racism and segregation within the organisation [3].

Calls to ‘decolonise’ global health by academics, practitioners, activists, and students differ in their approach and focus. While they jointly seek to address and dismantle racist and discriminatory structures and norms, few have delved into the more complex analysis of the “monopoly, misuse, or abuse” of power [4], including, crucially, the ways in which it is experienced.

Abuse–be it in the form of retaliation or rape, derogatory comments or discriminatory contracts, nepotism or neglect–stems from systemic asymmetries that foster, invite, and enable the misuse of power and dominance. Abimbola et al have explored facets of supremacy, encompassing coloniality, patriarchy, racism, white supremacy and saviorism, that together maintain power and privilege within global health [5]. These facets also underpin aid systems, resulting in internal inequalities that compound and spill over into how the mandate of an organization is implemented [6]. Discussions at MSF have centred, for example, on its two-tiered staff structure and the glaring over-representation of the “Global North” in management and governance [7, 8], more than 16 years after it committed to equal opportunities for all staff [9].

As Decolonise MSF organisers, we contributed to urgent demands for structural reform and transparency by publishing *Dignity at MSF* in September 2021, a movement-wide report on abuse and discrimination based on findings of an online survey with 359 current and former staff and stakeholders [10]. It is the first volunteer and survivor-led initiative to publicly assess and disclose findings on these subjects and the first to involve MSF’s global and historical staff base, as well as community partners.

MSF’s external accounting on abuse focuses on a relatively small number of formal complaints logged each year with ethics and behavioral units [11]. Our results present a startling contrast to this narrative. One in two MSF respondents (54.0%) witnessed or experienced one or more types of abuse (including discrimination) between July 2020 and July 2021. A majority (59.4%) have at one point reported abuse when reporting to line management is included. However, of these respondents, only 8.7% indicated feeling fully satisfied with outcomes, with over half (54.0%) believing that perpetrators faced no consequences at all. Respondents described personal and professional consequences for reporting; system weaknesses and perceived inaction left many with disappointment, distrust, and disdain for MSF itself. Cumulatively, our data suggests abuse is occurring at a significant scale and points to fundamental distrust in both the organisation’s accountability systems, and leadership’s ability to address this crisis.

These results are difficult, if not impossible, to contextualize because aid organisations rarely measure–or disclose–abuse or discrimination perceived by their workforce. Often there is no baseline: 2020 marked the first in MSF’s 50-year history in which it compiled formal complaints from global offices in addition to those from medical projects in the field [11]. As recently as October 2021, an institutional response to a racial justice questionnaire by *The New Humanitarian* stipulated that MSF lacks data for the one-year period since May 2020 on changed human resources practices and does not report on the percentage of leadership coming from under-represented groups [12].

In a context of inaccessible information on matters of the public interest, effective activism may require the direct production of research, even with limited means and methodological weaknesses, to advance understanding and future action when an institution cannot, or will not, study these questions. Such research may therefore be considered inherently agitational, requiring a mutual responsibility for diligence and safety by organisations and activists. Our report–created with free resources and volunteer labour–openly acknowledges its limitations, including possible self-selection despite our expansive outreach, ambiguities in question phrasing, our inability to verify employment status, and the lack of a suitable peer reviewer. We were not officially authorized to do this work but chose to disclose our authorship to promote full transparency and feedback. We nonetheless had to carefully navigate a maze of painstaking efforts to minimize our own legal and professional risks, including attacks by current MSF stakeholders and continual revision to ensure our report wording could not be framed as defamatory.

This type of research forces us to query the assumptions coded into the roles of *the researcher* and *researched*. Critically engaged activist research “provides an important approach to addressing the practical and ethical dilemmas of knowledge production” [13]. It confronts us with how we–as academics, activists, and practitioners–may dislocate ourselves from the problems we seek to change by looking forever outside rather than within, leaving us blind to how the same systems, hierarchies, and problems entangle and trap us from change [14].

As survivors of abuse within the organisation, we would argue that our lived experience–our entanglement–allowed us to formulate specific questions currently not captured by the institution, gain necessary trust, and better level power imbalances intrinsic to research processes. Our reflexivity served a process to unknot how power manifests towards and through us as individuals and institutions, resulting in a re-examination of the classical research model itself. Global health transformation requires relatable and personal discourse through reflexive dialogue between individuals across geographic, economic, and epistemic divides [15]. The report’s emphasis on storytelling, artwork, and counter-perspectives is consistent with this view.

As former staff with racialized backgrounds employed on international contracts, we have tried to embrace our “responsibility to speak up commensurate to position and privilege” [16]. Our report attempts to challenge institutional resistance by showcasing the pressing need for additional investigation, including a global independent assessment engaging survivors and patients, the vast majority of whom exist outside MSF’s associative and consultative structures. We hope it will also support fellow resistors at Decolonise MSF in tracing a path forward for future research and grassroots action.

If it succeeds, *Dignity at MSF* represents activist research in the spirit of the “generative power of protest”, which recognizes the “power of protest events themselves to shape internal movement dynamics” [17]. In our case, the vision is a movement for anti-racism and anti-discrimination at MSF that is self-sustaining, locally-led, heterogenous, and diverse in opinion and approaches. This requires the creation of a space where everyone can safely speak out and act. In crafting this report, we highlight the urgent need to reduce the structural barriers that contribute to the stifling of agency in pursuit of discovery–a process central to the long-term march to justice and equity in aid and global public health.
