# Correction: Research as agitation: Generative activism in the age of resistance

**DOI:** 10.1371/journal.pgph.0002658

**Published:** 2023-11-17

**Authors:** The PLOS Global Public Health Editors

## Correction

With this notice, we provide the following correction of [1]:

The article’s Competing Interests statement is updated to: As is noted in the article, AM and MM are former Medecins Sans Frontieres (MSF) employees, have been involved in the Decolonise MSF movement, and authored the ‘Dignity at MSF’ report cited as Reference 10.

### Additional Information about the ‘Dignity at MSF’ report [2]

This Opinion article [1] was commissioned by the Editors-in-Chief of *PLOS Global Public Health* in follow-up to the ‘Dignity at MSF’ report [2] (cited as reference 10 in [1]). After the Opinion [1] was published, a reader with an affiliation to MSF raised questions to PLOS about the strength of evidence to support claims in [1] due to concerns about the survey reported in [2]; the report in [2] is cited several times to support points made in [1].

PLOS followed up on the concerns raised, and the *PLOS Global Public Health* Editors concluded that the Opinion article is valid and meets the journal’s publication criteria.

The authors of [1] clarified that the survey reported in [2] stemmed from a volunteer-led initiative to gather, assess, and summarize personal accounts of self-reported abuse and discrimination in MSF. Care was taken with regard to informed consent, anonymization, and privacy, but the work was not designed or conducted as a formal research study and as such did not follow all established standards for human subjects research. Specifically:

- Institutional ethics approval was not obtained. The authors considered that it would not be feasible to obtain approval from MSF due to potential conflicts of interest.
- The survey tool was not formally validated. It was based on aims of preliminary exploration and was tailored to delve into issues of abuse and discrimination within MSF.
- Due to its exploratory purpose, participants for the ’Dignity at MSF’ survey were identified through an outreach effort involving current and former MSF staff, stakeholders, and community members without explicit exclusion criteria. (See also pages 12–14 of [2].)
